# Monocytic Myeloid-Derived Suppressor Cells in Chronic Infections

**DOI:** 10.3389/fimmu.2017.01895

**Published:** 2018-01-04

**Authors:** Anca Dorhoi, Nelita Du Plessis

**Affiliations:** ^1^Institute of Immunology, Bundesforschungsinstitut für Tiergesundheit, Friedrich-Loeffler-Institut (FLI), Insel Riems, Germany; ^2^Faculty of Mathematics and Natural Sciences, University of Greifswald, Greifswald, Germany; ^3^Department of Immunology, Max Planck Institute for Infection Biology, Berlin, Germany; ^4^Division of Molecular Biology and Human Genetics, Department of Biomedical Sciences, Faculty of Medicine and Health Sciences, SAMRC Centre for Tuberculosis Research, DST and NRF Centre of Excellence for Biomedical TB Research, Stellenbosch University, Tygerberg, South Africa

**Keywords:** myeloid-derived suppressor cells, infection, inflammation, tuberculosis, human immunodeficiency virus, *Staphylococcus*, viral hepatitis

## Abstract

Heterogeneous populations of myeloid regulatory cells (MRC), including monocytes, macrophages, dendritic cells, and neutrophils, are found in cancer and infectious diseases. The inflammatory environment in solid tumors as well as infectious foci with persistent pathogens promotes the development and recruitment of MRC. These cells help to resolve inflammation and establish host immune homeostasis by restricting T lymphocyte function, inducing regulatory T cells and releasing immune suppressive cytokines and enzyme products. Monocytic MRC, also termed monocytic myeloid-derived suppressor cells (M-MDSC), are *bona fide* phagocytes, capable of pathogen internalization and persistence, while exerting localized suppressive activity. Here, we summarize molecular pathways controlling M-MDSC genesis and functions in microbial-induced non-resolved inflammation and immunopathology. We focus on the roles of M-MDSC in infections, including opportunistic extracellular bacteria and fungi as well as persistent intracellular pathogens, such as mycobacteria and certain viruses. Better understanding of M-MDSC biology in chronic infections and their role in antimicrobial immunity, will advance development of novel, more effective and broad-range anti-infective therapies.

## Introduction

Mononuclear myeloid cells encompass various phagocyte populations exerting distinct functions during infection. From progenitors and immature myeloid cells (IMC) to mature and polarized phagocytes, subsets of myeloid regulatory cells (MRC) have been described. These populations include regulatory dendritic cells (DCs), regulatory and alternatively activated macrophages (M2-like macrophages), tumor-associated macrophages (TAM), and a unique mixture of heterogeneous cells coined myeloid-derived suppressor cells (MDSC) (1). This nomenclature indicates their origin and ability to suppress T-cell immunity (2). MDSC comprise morphologically distinct subsets, monocyte-like [monocytic MDSC (M-MDSC)] and neutrophil-like (PMN-MDSC) cells. Phenotypically, M-MDSC are HLA-DR^−/low^CD11b^+^CD33^+/high^CD14^+^CD15^−^ in humans and Gr-1^dim/+^CD11b^+^Ly6C^+^Ly6G^−^ in mice (2). Several studies report on CD11b^+^Ly6C^+/dim^Ly6G^int^ murine M-MDSC, a phenotype that requires further validation in additional disease models and in-depth characterization (3, 4). These cells have biochemical features characteristic of the myeloid lineage, notably abundance of products downstream of arginase 1 (ARG1), inducible nitric oxide synthase (iNOS), indoleamine dioxygenase (IDO), and cyclooxygenase (COX1) (2, 5). Unequivocal phenotypic markers for MDSC have not been identified so far, implying that cells can only be classified as MDSC upon demonstration of their lymphocyte suppressive function. This suggests that MDSC are likely underreported, particularly in conditions characterized by expansion of myeloid cells such as in infectious diseases.

Most of the information on MDSC emerges from cancer research where MDSC are associated with poor disease outcome. However, reports on myeloid suppressor cells in infection date back four decades. “Natural suppressor” cells were identified in spleens of experimentally infected animals following systemic delivery of mycobacteria, notably the vaccine strain *Mycobacterium bovis* Bacille Calmette–Guérin (BCG) (6). Although research on suppressor cells in cancers has flourished since then, studies in infectious diseases lagged behind. Cancer and infection share several pathophysiological features, including the non-resolving inflammation (7), which often triggers emergency hematopoiesis and expansion of MDSC (8). Given such similarities and encouraged by progress made in cancer biology, recent investigations found MDSC in communicable diseases (9–12), uncovered their interactions with microbes and emphasized critical roles in disease pathogenesis. This review focuses on M-MDSC and discusses their genesis during infection as well as interactions with immune cells, elaborating on targets and mechanisms of suppression. We will mostly describe M-MDSC biology in infections caused by *M. tuberculosis, Staphylococcus aureus*, hepatitis viruses [hepatitis B virus (HBV), hepatitis C virus (HCV)], and human immunodeficiency viruses (HIV) and to a lesser extent fungi and parasites (Box 1). We will use the term MDSC to refer to the total MDSC population, without further subset phenotype characterization. For studies using monocytic subsets, within the MDSC pool, we will use the acronym M-MDSC.

Box 1 **Chronic infections associated with monocytic myeloid-derived suppressor cells (M-MDSC).**

Monocytic myeloid-derived suppressor cells have been reported in various infections caused by bacterial and viral agents, many of them causing diseases highly relevant for the public health. Key points about the pathogen and the respective disease are presented in the following. *M. tuberculosis* is a Gram-positive bacterium and represents the etiologic agent of human tuberculosis (TB). TB primarily affects the lungs of millions of people, and is among the top 10 causes of death worldwide (13). Infection with *M. tuberculosis* frequently leads to latent TB, bacteria being contained within tissue lesions, but not eliminated. Such individuals, estimated at one-third of global population, are at risk of developing active TB upon immune suppression. *S. aureus* is a Gram-positive bacterium that often colonizes the human skin and nose (14). It is the leading cause of skin and soft tissue infections, pneumonia, osteomyelitis, endocarditis, and septicemia. Such conditions can manifest as acute and often long-lasting, frequently nosocomial-associated diseases, which are often resistant to antibiotics. Increased antimicrobial resistance characterizes current clinical isolates of *M. tuberculosis* and *S. aureus*. This results in significant therapy failures and economic burdens because of refractoriness to canonical chemotherapy (15). HCV and HBV are single-stranded RNA (*Flaviviridae*) and double-stranded DNA (*Hepatdnaviridae*) viruses, respectively, which cause chronic infection of the liver leading to end-stage liver disease in the absence of therapy. Prevalence of HCV and HBV in human population is high, reaching 70 million and 250 million chronic cases, respectively (16). HIV, encompassing HIV-1 and HIV-2, are lentiviruses belonging to the *Retroviridae* family that cause the acquired-immune deficiency syndrome (AIDS). AIDS affects more than 35 million people worldwide and the virus causes lytic infection of immune cells, primarily CD4+ lymphocytes (17). Often AIDS leads to reactivation of latent TB and such a comorbidity results in high death tolls (13).

## Genesis of M-MDSC in Infectious Diseases

**Figure 1:**
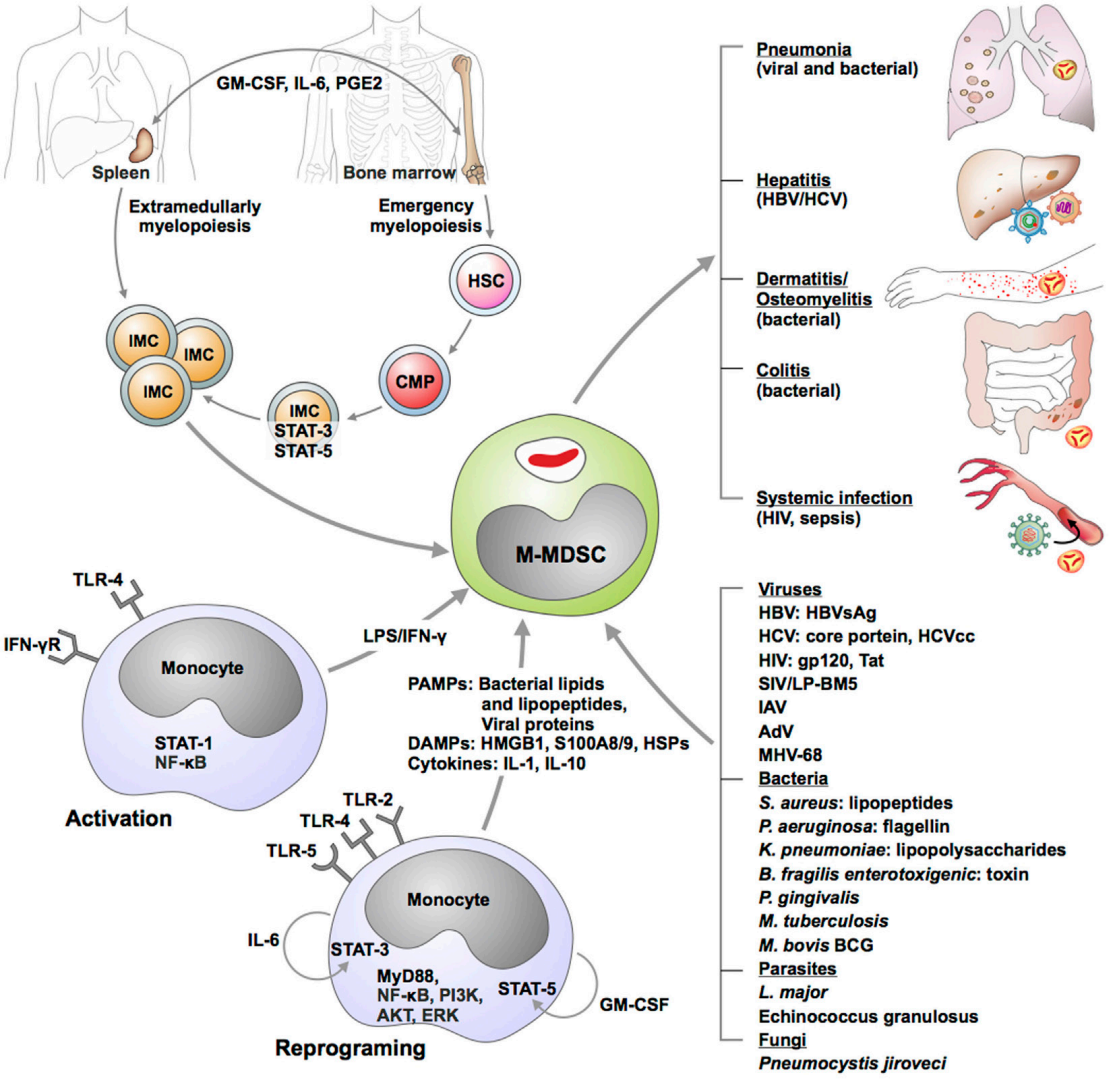
Genesis of monocytic myeloid-derived suppressor cells (M-MDSC) during infectious diseases. Hypothetical models were derived from *ex vivo* results, correlative studies in animal models as well as clinical observations. Immature myeloid cells (IMC) are generated either in bone marrow or in spleen as a consequence of emergency myelopoiesis. Growth factors, cytokines, and lipids promote progression of hematopoietic stem cells (HSC) toward common myeloid progenitor (CMP) development and subsequent IMC genesis. Combination of cytokines as well as direct stimulation of selected microbial receptors by various microorganisms may activate or reprogram circulating monocytes toward M-MDSC. M-MDSC are recruited in various organs where they exert suppressive function and modulate manifestations and outcome of the disease. Abbreviations: AdV, adenovirus; AKT, protein kinase B; ERK, extracellular signal-regulated kinase; GM-CSF, granulocyte-macrophage colony stimulating factor; gp120, glycoprotein 120; HBV, hepatitis B virus; HBVsAg, HBV soluble antigen; HCV, hepatitis C virus; HIV, human immunodeficiency virus; IAV, influenza A virus; IFN-γ, interferon gamma; IL-6, interleukin 6; LPS, lipopolysaccharide; LP-BM5, virus murine acquired-immune deficiency syndrome (AIDS); MHV-68, murine herpesvirus 68; MyD88, myeloid differentiation primary response gene 88; NF-κB, nuclear factor “kappa-light-chain-enhancer” of activated B-cells; PI3K, phosphatidylinositide 3-kinase; PGE2, prostaglandin E2; STAT, signal transducer and activator of transcription; SIV, simian immunodeficiency virus; tat, trans-activator of transcription; TLR, toll-like receptor.

Expansion of M-MDSC occurs in various infectious diseases. Accumulating evidence indicate that oncogenic viruses, including HBV (18) and HCV (19–22), retroviruses, notably HIV (23, 24), simian immunodeficiency virus (SIV) (25, 26), and mouse immunodeficiency virus LP-BM (27), as well as Gram-positive bacteria, such as mycobacteria (28–30), staphylococci (31–33), enterotoxigenic bacilli (34), and Gram-negative pathogens, such as klebsiellae (35), trigger generation of M-MDSC. Fluctuation of this MDSC subset during anti-infective therapy was demonstrated in patients undergoing canonical TB chemotherapy (29), further strengthening the notion that disease progression in chronic infections is associated with expansion of M-MDSC. For some microbes, precise microbial cues and corresponding host pathways triggering M-MDSC generation or reprogramming of monocytes into M-MDSC have been elucidated (Figure 1). However, to date, for most infections, expansion of M-MDSC is explained solely by generation of inflammatory mediators during the course of the disease. Cytokines (IL-1 family members, IL-6, TNF, IL-10), lipid mediators (prostaglandin E2, PGE2), and growth factors (GM-CSF) foster generation of M-MDSC by promoting emergency myelopoiesis, skewing differentiation of progenitors into monocytes and DCs (STAT3/STAT5 activation) and promoting survival of M-MDSC (TGF-β, MCL-1-related anti-apoptotic A1) (36–40) (Figure 1). Just like in cancer, M-MDSC and populations containing M-MDSC are detectable at the site of pathology; e.g., in infected lungs in TB (29, 30, 41), pneumonia caused by *Francisella tularensis* (42), and influenza A virus (43, 44), in liver during HBV infection (45, 46), in skin and prosthetic bone implants during *S. aureus* colonization (32, 47, 48), and systemically in AIDS and sepsis (23, 24, 49). M-MDSC have also been detected in bone marrow and spleen, e.g., in TB (50), indicating their origin.

### Microbial Signatures and Microbial Sensors Trigger M-MDSC Genesis

#### Pathogen Sensors Involved in Generation of M-MDSC

Microbial signatures are detected by non-clonally distributed innate receptors termed pattern recognition receptors (PRR). PRR are grouped in families and the founder toll-like receptors (TLR) have been best characterized so far. TLR are present on the plasma membrane and within endosomes and are activated by diverse microbial structures, including lipids [e.g., TLR-4 senses lipopolysaccharide (LPS)], lipoproteins (e.g., TLR-2 senses acylated peptides) and proteins (e.g., TLR-5 senses flagellin). Generally, microbial-derived cognates of TLR-2 and -4 induce M-MDSC (20, 32, 51–53). LPS, which is the major cell wall component of Gram-negative bacteria, triggers proliferation of HSC (40) and induces M-MDSC upon pulmonary instillation or subsequent infection with *Salmonella* spp. or *Klebsiella pneumonia* (35, 51). Stimulation of human monocytes with TLR-4 agonists reprograms the cells into M-MDSC in a process dependent on STAT-3 activation (54). Crosstalk between TLR/MyD88 and JAK2/STAT5 pathways following receptor activation by LPS and GM-CSF is critical for M-MDSC generation (35, 51). The adaptor MyD88, which converges signals from multiple TLR, has also been implicated in generation of MDSC during polymicrobial sepsis (55). TLR-4 appears dispensable for sepsis-induced suppression of T cells (55) thereby indicating that IL-1, which binds IL-1R upstream of MyD88, conditions MDSC differentiation.

Several bacterial and viral agonists of TLR-2 promote M-MDSC differentiation from monocytes and in certain instances precise signaling pathways have been identified. *S. aureus* lipopeptides activate TLR2/6 dimers in skin cells for IL-6 production which in turn promote local MDSC accumulation (32). HCV reprograms monocytes into M-MDSC by stimulating TLR-2. More precisely, HCV core proteins or HCV cell culture-derived virions trigger TLR-2/PI3K/AKT/STAT3 pathway and this leads to cytokine production, notably IL-10 and TNF-α, and monocyte differentiation into MDSC (19–21). By contrast, TLR-3 ligation restricts HCV and LPS-induced M-MDSC differentiation (19, 52). Nonetheless, vesicular stomatitis virus activation of TLR-3 induces MDSC expansion (56). Alike TLR-3, TLR-7 activation by influenza virus blocks MDSC, including M-MDSC, accumulation in infected lungs (44). Both TLR-3 and -7 are located in endosomes. Whether signal compartmentalization, notably at the cell membrane or within endosomes, is critical for MDSC genesis remains to be established. Very little information exists on the roles of cytosolic PRR, such as nod-like receptors and AIM-like receptors, in monocyte reprogramming or M-MDSC generation. Moreover, many pathogens, notably mycobacteria, simultaneously stimulate multiple PRR (57) and the net outcome of such innate recognition on M-MDSC in TB awaits clarification.

Host alarmins that activate PRR have also been implicated in MDSC generation. S100A proteins, high-mobility-group-protein B1 and heat-shock proteins bind the receptor for advanced glycation products (RAGE), TLR-2, and TLR-4. In cancer and autoimmune diseases, these ligands have been associated with increased dynamics of MDSC, including M-MDSC (58–60). Just like microbial-derived PRR agonists, alarmins may induce cytokine release, such as IL-6 and subsequent autocrine or paracrine differentiation of immature mononuclear cells toward MDSC (61). In chronic infections, for instance, in TB patients, S100A8/9 proteins are abundant in the lung (62). These alarmins besides driving recruitment of MDSC (63) bind RAGE and subsequently upregulate ARG1, a key suppressive enzyme in M-MDSC (2). Since tissue damage often occurs during microbial insult, PRR stimulation by host-derived danger molecules along with microbial-derived agonists could contribute to the regulation of MRC. Similarly, synergy between microbial products, such as LPS, and inflammatory cytokines, notably IFN-γ, restricts differentiation of DCs and fosters genesis of M-MDSC in the bone marrow (64).

#### Microbial Factors Required for M-MDSC Genesis

For many microbes, the precise pathways required for M-MDSC genesis are not known. Mycobacteria induce accumulation of such cells irrespective of key virulence features, notably the type VII secretion system. M-MDSC have been reported for both *M. tuberculosis* and the vaccine BCG (29, 30, 50, 65). Mycobacterial glycolipids appear sufficient to induce these regulatory monocytes, as indicated by the presence of MDSC in animals inoculated with complete Freund’s adjuvant (66). In contrast to mycobacteria, non-colitogenic bacteria and oncogenic gut species (*Fusobacterium nucleatum*, pks^+^ *Escherichia coli*) do not trigger M-MDSC, whereas enterotoxigenic *Bacillus fragilis* employs the toxin to prime epithelial cells for IL-17 and M-MDSC expansion (34). HIV and SIV infection triggers accumulation of M-MDSC in the blood and their reduction in the bone marrow, which correlates with plasma viral loads and disease progression (25, 49). Several HIV viral factors promote expansion of the M-MDSC or reprogramming of monocytes. Human monocytes stimulated with HIV gp120 (23, 24) and/or with Tat proteins (54) acquire T-cell suppressive activity. This differentiation requires autocrine release of IL-6 and activation of STAT-3 (23, 54). HBV surface antigen similarly triggers differentiation of human monocytes toward M-MDSC in an autocrine manner depending on activation of the kinase ERK and the transcription factor STAT-3 (18). The necessity of specific kinases, such as ERK (18) and AKT (19, 20) for microbial-induced M-MDSC generation resembles kinase signatures of MDSC in cancer (67). Similarly, STAT-3 is required for M-MDSC in cancer (68) as well as during infection with HIV (23, 54), HCV (20, 22), and stimulation with bacterial LPS (54). For many bacterial (*Mycobacterium* spp., *F. tularensis, Porphyromonas gingivalis*) (29, 30, 42, 50, 69) and viral pathogens [vaccinia virus, lymphocoriomeningitis virus (LCMV), MCMV, murine gamma virus, LP-BM5] (70–72), and protozoa (*Leishmania* spp.)(73, 74), the host pathways or microbial signatures required for M-MDSC genesis are still undefined.

### Inflammation Drives M-MDSC Generation during Infection

A common denominator in infection and cancer biology is the inflammation. Whereas physiological inflammation protects the host and restores homeostasis, in exuberant acute infections and chronic processes, inflammation often becomes pathologic and leads to disease manifestation. In such a scenario, inflammation-induced pathology becomes life-threatening. M-MDSC are primarily associated with chronic infections; however, they have been also reported in acute infectious diseases. Genesis of this myeloid regulatory subset is uncoupled from a specific phase of an infectious process. For instance, *F. tularensis* triggers IMC with M-MDSC features during acute, but not sub-acute, non-lethal infection (42). In polymicrobial sepsis M-MDSC are present early, as well as at late stages of sepsis, during the suppressive phase (55, 75). In infection with the LCMV, acute strains (Armstrong) do not induce M-MDSC, whereas chronic strains (Clone 13) induce suppressive myeloid cells (71).

Certain transcription factors and inflammatory mediators are critical for generation of MRC in infections. These requirements resemble those observed for MDSC in cancer (63). In sepsis, myeloid specific deletion of the myeloid differentiation-related transcription factor nuclear factor I-A, or deletion of the transcription factor C/EBPβ, result in reduction of MDSC, including M-MDSC (76, 77). Pro-inflammatory cytokines, notably IL-6, TNF-α, and IL-1, drive generation of MDSC in various infection models. In viral infections, including HIV (23) and HBV (18), IL-6 reprograms monocytes into suppressor cells. The same cytokine drives accumulation of M-MDSC in *S. aureus* skin infection and into the lungs subsequent to LPS instillations (32, 35). TNF promotes differentiation of MDSC in chronic inflammation (37, 78), likely through membrane expression of TNFR2, as shown in sterile inflammation (79). TNF signaling contributes to M-MDSC generation in HCV infection (19) and regulates M-MDSC dynamics and activity also in murine mycobacterial infection (80). Besides cytokines, pro-inflammatory lipids such as the eicosanoid PGE2 are highly abundant in the TB-susceptible mouse strain C3HeB/FeJ (81) and these animals also accumulate M-MDSC (41). Interestingly, application of a COX2 inhibitor which lowers PGE2 levels rescues C3HeB/FeJ from TB lethality (81), thereby suggesting that this lipid may be critical for genesis of host-detrimental MDSC in TB. In addition, PGE2 positively regulates enzymatic pathways critical for the suppressive function of the MDSC, including iNOS, IDO1, and IL-10. COX2 crosstalks with the IL-1/IL-1R pathway, as well as with IFN I pathway, which has been revealed in TB and flu (82, 83). The positive cross-regulation between COX2 and IL-1 may affect M-MDSC genesis. IL-1/IL-1R pathway drives accumulation of M-MDSC in BCG-vaccinated mice (65). IL-1β also regulates PMN-MDSC generation by itself and during fungal disease (84). Activation of specific inflammasomes for release of bioactive IL-1β has not yet been related to MDSC induction during infectious diseases. However, the NLRP3 inflammasome drives MDSC accumulation in cancer (85). To what extent key inflammatory molecules, including IL-1β and the downstream inflammasome platforms, may affect generation and accumulation of M-MDSC in other chronic infections than TB remains to be established.

As a corollary, various stimuli trigger M-MDSC generation and expansion during microbial insult. Additional pathways will likely be uncovered as the research into M-MDSC in infection expands. Recent studies indicate that GM-CSF licenses monocytes for suppressive activity upon further stimulation with PRR agonists or cytokines (86). Such a two-step process likely occurs during infection. Furthermore, fate-mapping studies are imperative to elucidate whether bone marrow or extramedullary myelopoiesis are unique sites for M-MDSC expansion or whether this myeloid subset can self-maintain *in situ*, at the site of the infection. Furthermore, the signals triggering recruitment of M-MDSC at the site of the pathology require further elucidation. Panoply of chemokines and alarmins are generated during infection. These, along with factors known to drive MDSC accumulation in cancer may be essential for MDSC dynamics in infected tissue. For instance, both PGE2 and TGF-β upregulate CXCR2 and CXCR4 expression in M-MDSC in cancers and they may be critical for the accumulation of such cells toward CXCL12 or CCL2 gradients at the site of infection, as it has been demonstrated in tumors (63, 87–89).

## M-MDSC in Pathophysiology of Chronic Infections

### M-MDSC Immunosuppressive Mechanisms and Cellular Interactions

**Figure 2:**
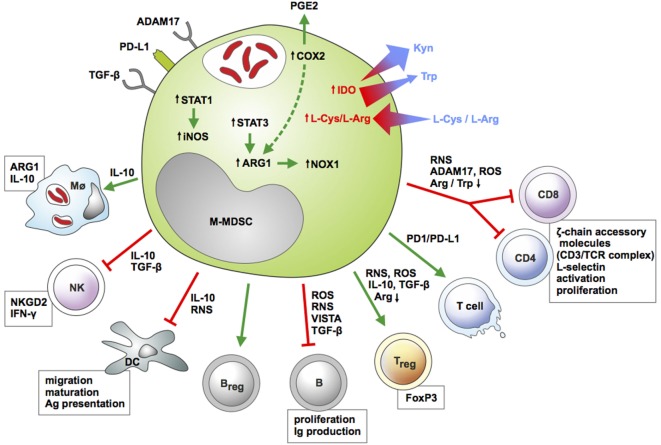
Features of monocytic myeloid-derived suppressor cells (M-MDSC) and their interactions with immune cells during infection. M-MDSC express membrane-bound inhibitory receptors and upregulate enzymatic pathways [inducible nitric oxide synthase (iNOS), ARG1, COX2, IDO] conferring suppressive activity toward multiple myeloid and lymphoid cell subsets. The key function of M-MDSC is suppression of T-cell immunity. M-MDSC restrict proliferation and release of cytokines by effector CD4 and CD8 lymphocytes and induce apoptotic cell death in these cells. In addition, these myeloid regulatory cells induce regulatory T and B cells, while limiting antibody release and proliferation of conventional B cells. M-MDSC alter activity of NK cells and antigen-presenting cells (APCs) and induce polarization of macrophages toward a regulatory phenotype. Color-coded arrows indicate induction/activation (green) or suppression (red), and molecules employed by M-MDSC for such effects are highlighted. Size- and color-coded arrows indicate gradient fluxes for selected essential amino acids. Boxes indicate cellular functions or pathways modulated by M-MDSC. Abbreviations: ADAM17, ADAM metallopeptidase domain 17; ARG1, arginase 1; CD, cluster of differentiation; COX2, cyclooxygenase 2; DC, dendritic cell; IDO1, indoleamine dioxygenase 1; IFN-γ, interferon gamma; IL-10, interleukin 10; iNOS, inducible nitric oxide synthase; Kyn, kynurenine; l-Arg, l-arginine; l-Cys, l-cysteine; MΦ, macrophage; NK, natural killer cell; NKGD2, killer cell lectin like receptor K1; NOX1, NADPH oxidase 1; PGE2, prostaglandin E2, PD-L1, programmed-death ligand 1; RNS, reactive nitrogen species; ROS, reactive oxygen species; STAT, signal transducer and activator of transcription; TGF-β, transforming growth factor beta; Trp, tryptophan; VISTA, V-domain Ig suppressor of T-cell Activation.

Myeloid regulatory cells regulate host immunity through interaction with immune and non-immune cells (90) (Figure 2). This link is typically bi-directional: e.g., T-cells also regulate MRC expansion and activity, to induce tissue healing and remodeling (91, 92). Here, we describe current information on monocytic MDSC immunosuppressive machinery and interaction with archetypal immune cells (Table 1).

**Table 1:** Impact of monocytic myeloid-derived suppressor cells (M-MDSC) on infectious disease outcome and their immunosuppressive effects.

Microbial organism	Context of M-MDSC investigation	Major outcome; immunosuppressive effect	Reference
**Viruses**			
Immunodeficiency virus [human immunodeficiency viruse (HIV), simian immunodeficiency virus, LP-BM5]	M-MDSC and total MDSC	Host detrimental; suppress T-cell and B-cell responses, express inducible nitric oxide synthase (iNOS), and produce reactive oxygen species (ROS), ARG-1, IL-10, induce Treg	Gama et al. (26); Vollbrecht et al. (49); Qin et al. (24); Green et al. (93); Garg and Spector (23); Sui et al. (94); Wang et al. (54); O’Connor et al. (95); du Plessis et al. (28); Sui et al. (25); Garg et al. (96); Dross et al. (97)

Cytomegalovirus (CMV)	M-MDSC-like	Host detrimental; impair T-cell expansion, slowing viral clearance	Daley-Bauer et al. (70)

Hepatitis C virus (HCV)	M-MDSC and total MDSC	Host detrimental; suppress CD4 T-cell and NK cell function, increase Treg	Tacke et al. (21); Salem et al. (98); Zeng et al. (99); Nonnenman et al. (100); Ning et al. (101); Goh et al. (102); Ren et al. (22); Lei et al. (103); Pang et al. (19); Ren et al. (104)

Hepatitis B virus (HBV)	M-MDSC and total MDSC	Host detrimental; express IL-10, suppress T-cell function, promote disease chronicity	Chen et al. (45); Huang et al. (105); Kondo et al. (106)

Viral coinfection (HIV/CMV, HCV/HIV)		Host detrimental; impair T-cell function, accelerate disease progression	Lei et al. (103); Garg et al. (96); Tumino et al. (107)

**Bacteria**			
*Staphylococcus aureus*	M-MDSC and PMN-MDSC	Host detrimental; suppress T-cell function, express ARG-1, iNOS, IL-10, exacerbate disease, promote disease chronicity	Skabytska et al. (32); Heim et al. (108); Heim et al. (47, 48); Tebartz et al. (33); Peng et al. (31)

*Francisella tularensis*	Total MDSC	Host detrimental; reduced phagocytosis, reduced survival	Periasamy et al. (42)

*Mycobacteria* spp.	M-MDSC and total MDSC	Host beneficial/detrimental; suppress T-cell function; express ARG-1 and iNOS, impaired pathogen killing; TNF-dependent suppression of CD4 T cells	Dietlin et al. (109); Martino et al. (65); Obregón-Henao et al. (41); Knaul et al. (30); Tsiganov et al. (50); Yang et al. (110); du Plessis et al. (28); Chavez-Galan et al. (80)

*Klebsiella pneumoniae*	M-MDSC and PMN-MDSC	Host beneficial/detrimental; pro-resolving, express ARG-1, IL-10/impair phagocytosis/killing	Poe et al. (35); Ahn et al. (3); Chakraborty et al. (4)

*Helicobacter pylori*	M-MDSC	Host detrimental; suppress protective TH1 development.	Zhuang et al. (111)

Polymicrobial sepsis	M-MDSC and total MDSC	Host beneficial/detrimental; suppress T-cell function, express nitric oxide and pro-inflammatory cytokines (early) and ARG-1, IL-10, and TGF-β (late)	Delano et al. (55); Sander et al. (112); Brudecki et al. (75); McPeak et al. (76, 77)

*Escherichia coli*	M-MDSC	Host detrimental; suppress T-cell activation, innate immunity, impair bacterial uptake and increase disease severity, infection susceptibility	Bernsmeier et al. (52)

**Protozoa**			
*Leishmania* spp.	M-MDSC and total MDSC	Host beneficial/detrimental; species-specificity, suppress CD4 T-cell proliferation, improved killing of parasites	Pereira et al. (73); Schmid et al. (74); Ribeiro-Gomes et al. (113); Bandyopadhyay et al. (114); Hammami et al. (115)

*Trypanosoma cruzi*	M-MDSC and PMN-MDSC	Host beneficial/detrimental; dependent on MDSC subset, express ROS, NO, suppress CD8 T-cell proliferation	Goni et al. (116); Cuervo et al. (117); Arocena et al. (118)

*Toxoplasma gondii*	Total MDSC	Host protective; express NO, control parasite replication	Voisin et al. (119); Dunay et al. (120)

**Helminths**			
*Schistosoma* spp.	Total MDSC	Not evaluated; express ROS, suppress T-cell responses	Yang et al. (121)

*Echinnococcus granulosus*	Total MDSC	Not evaluated; association with increased Treg and impaired T-cell L-selectin	Pan et al. (122)

*Nippostrongylus brasiliensis*	M-MDSC and PMN-MDSC	Host beneficial/detrimental; dependent on MDSC subset, express TH2 cytokines, reduce parasite burden (PMN-MDSC)	Saleem et al. (123)

*Heligmosomoides polygyrus bakeri*	Total MDSC	Host detrimental; suppress CD4 T-cell proliferation, increase parasite burden, and promote chronic infection	Valanparambil et al. (124, 125)

*M-MDSC are studied as a purified cell population or as part of the total MDSC population to measure their impact on the host control of infectious pathogens*.

#### T Cells

Immunosuppression by MDSC has the potential to inhibit innate and adaptive immune cell activation, proliferation, viability, trafficking, and cytokine production. M-MDSC utilize a variety of suppressive mechanisms and likely differ in their ability to initiate antigen-specific versus non-specific suppression (126, 127). Each immune suppressive function is determined by the type of MRC, the microenvironmental components and the state of T-cell activation, favoring the probability that non-specific and antigen-specific suppressive mechanisms may coincide. Although not the focus of this review, as an example, PMN-MDSC can present peptides to T cells, but their low expression of major histocompatibility complex (MHC) II and costimulatory molecules, suggest they might only affect CD8 T-cell responses in an antigen-specific manner, as reported during retrovirus infection (128). This idea is supported by reports on MDSC-mediated inhibition of antigen-specific CD8 T-cell responses in tumors, likely due to the MHC I-restricted nature of cancer MDSC (2, 127, 129, 130). In infection, antigen-specific immunosuppression of CD8 T cells by M-MDSC is restricted to polymicrobial sepsis (131), HCV (21), HBV (46), murine encephalomyelitis virus (132), SIV and HIV infections (26), and LCMV infection (71). Data on the effect of MDSC on CD4 T helper cell (TH) subsets during infectious diseases are limited, but do exist as a result of the MHC-independent suppressive effects of MDSC in the context of HCV (21), HIV (24), and murine encephalomyelitis virus infection (132). During BCG-induced pleurisy, transmembrane TNF on M-MDSC restricts proliferation of CD4 T cells *via* interaction with lymphocyte-expressed TNFR2 (80). Results on MDSC interaction with TH17 and TH2 polarized CD4 T cells are contradictory and reports exist of mainly PMN-MDSC-mediated induction and suppression of TH17 responses in cancer, autoimmunity and infection (133–138), likely indicating that the combination of mediators present in the microenvironment determines the final outcome. In turn, TH1 and TH2 are involved in the expansion and activation of MDSC in cancer and also hepatitis (137, 139). Interestingly, recent findings suggest that CD1d-restricted natural killer T cells can convert immunosuppressive murine-MDSC into immune stimulating APCs following influenza virus infection, *via* their interaction with CD40 (140).

Regulatory T cells (Treg) are equally important components of the host immunoregulatory network. Data suggest reciprocal regulation of MDSC and Treg through mechanisms involving presence of IL-10, TGF-β, IL-4Rα, p47phox, PD-L1, TGF-β, and CD40–CD40L interactions, ARG1 induction and CCR-5-mediated recruitment (91, 126, 141–144). Interactions between total MDSC and Treg in cancer are well described (145, 146) with Treg depletion reducing MDSC immunosuppression by lowering their expression of PD-L1 and IL-10 production (147). Evidence of interaction in non-cancerous models, including type-1 diabetes, cardiac allograft and airway hyper-responsiveness, also exist (148–150). More specifically, the induction of Treg by M-MDSC, has also been described during HIV infection and shown to contribute to host immunosuppression (23, 49, 54). Data by O’Connor suggest reciprocal crosstalk between M-MDSC and Treg during LP-BM5-induced murine AIDS. Here, M-MDSC subsets display differential suppression of T- and B-cells, thereby indicating functionally overlapping, but distinguishable, immunosuppressive effects (27, 95). Incubation of M-MDSC from peripheral blood of HIV-1-infected individuals, even those on antiretroviral therapy with undetectable viremia, with CD4 T cells from healthy individuals, significantly increased differentiation of Foxp3 Treg, whereas depletion of MDSC significantly increased IFN-γ production by CD4 T cells (54).

#### B Cells

Information on MDSC interaction with B-cells only recently started to accumulate. In autoimmune disease, M-MDSC inhibit B-cell proliferation and antibody production *via* an iNOS and a PGE2-induced pathway (151). However, opposing data demonstrated that the total MDSC population promotes proliferation and differentiation of immunoglobulin-A-producing immunosuppressive plasma B-cells *via* cell contact in mouse tumor models (152). In infectious diseases, M-MDSC suppressed B-cell responsiveness to retroviral infection in mice *via* iNOS and the negative immune checkpoint regulator V-domain Ig Suppressor of T-cell Activation (VISTA) (72, 93).

#### Myeloid Cells

Data on MDSC interaction with myeloid cells, such as DC, neutrophils, and macrophages in infectious diseases, are equally restricted, with reports mainly revealing that their inhibitory effects are exacerbated by cross-regulation with macrophages at tumor sites. In lung infections, such as *Pneumocystis* pneumonia (PcP), M-MDSC expressing PD-L1 are induced and impair alveolar macrophage (AM) phagocytic activity while increasing AM expression of PD-1 (153). MDSC interaction with neutrophils has been described in mice infected with *K. pneumoniae* or challenged with LPS, demonstrating that MDSC efferocytose infected, apoptotic neutrophils (35). Furthermore, M-MDSC suppress DC maturation, antigen uptake, migration, and TH1 cytokine production following administration of a DC vaccine for malignant melanoma (154). Similar findings were reported following LPS stimulation and in hepatocellular carcinoma, where both MDSC subsets reduced expression ofMHC II, stimulatory molecules on DC, and cytokine production (64, 155). It stands to reason that these MDSC-induced modifications, affecting DC-mediated activation of T cells and antigen uptake, could also be effective in infectious diseases and warrant further investigation.

#### Natural Killer (NK) Cells

Reports on MDSC-mediated impairment of NK cell function emanate mainly from the cancer field. NK cells are critical to the innate immune system, exhibit cytotoxic and cytolytic functions, and target pathogens and malignant cells. In tumors, M-MDSC and also a population containing M-MDSC, inhibit cytotoxic activity and cytokine production by NK cells through cell contact-dependent mechanisms involving membrane-bound TGF-β and NKp30 ligand (156–158). NK cell-mediated suppression by total HLA-DR^lo^CD33^+^CD11b^lo^ MDSC has also been reported in chronic HCV infection and it is mediated *via* an ARG1-dependent inhibition of mammalian target of rapamycin (102).

### Kinetics, Interference with Immunity, and Impact on Disease Outcome

The immune inhibitory functions of M-MDSC have extensive consequences on disease outcome (Table 1). According to current understanding, the class of pathogen and the immune mediators present, collectively determine pathogen persistence versus clearance. M-MDSC have versatile roles in infection, with either beneficial or detrimental outcomes for the host depending on the pathogen and the course of infection. During long-lasting infections, MDSC may even exhibit dual roles depending on the disease stage. E.g., M-MDSC are host-protective in certain fulminant acute infections by restricting immunopathology (35, 112, 159). During late sepsis, the immature total MDSC population aggravates disease (76, 77, 160). M-MDSC may, however, be harmful in acute infection with intracellular microbes, notably francisellae (42). Alternatively, M-MDSC may be detrimental to the host, irrespective of the phase of the disease, as reported in AIDS (25). By limiting anti-viral immunity early, these regulatory monocytes foster disease progression, while provoking disease exacerbation during the chronic HIV infection.

#### Viruses

Viral infections are known for their induction of pro-inflammatory mediators associated with the generation of MDSC. E.g., M-MDSC are increased in both clinical and experimental viral infections, such as HIV, SIV, and LP-BM5 (25–27, 49, 93, 94). During these retroviral infections, increased levels of M-MDSC are likely detrimental to disease outcome and facilitate pathogen survival, when considering the TH1 immunosuppressive effect and correlation to viral load and CD4 T-cell count (24, 49, 54, 95). Interestingly, HIV infection-mediated expansion of M-MDSC in peripheral blood mononuclear cells may also negatively affect containment of other concurrent infections, as reported for cytomegalovirus (CMV) infection (96). Recruitment of M-MDSC-like cells were also reported for murine CMV mono-infection and shown to impair viral clearance (70). Information on MDSC in HCV infections has been variable, but largely provides evidence of unfavorable effects on host protective immunity (19, 22, 104). Increased MDSC frequencies positively correlate with HCV viral load and decreased CD8 T-cell function (21, 99). Reports show that elevated levels of immature Lin^−^HLA-DR^−^CD33^+^CD11b^+^ MDSC, consisting of M-MDSC and PMN-MDSC, in chronic HCV-infected patients, decline following successful IFN-α treatment (98), while treatment-naive HCV-infected individuals show significantly increased liver- and circulating MDSC frequencies compared to treated and uninfected individuals (99, 161). Nonetheless, other *in vivo* investigations failed to show significant MDSC elevations or an association with viral load (100). Ning et al. also provided evidence of increased M-MDSC in HCV-infected patients; however, this was correlated with age and not viral load, suggesting that the immune response caused by viral replication, rather than the virus itself, is responsible for increased M-MDSC (101). HBV infections are also associated with induction of MDSC. HLA-DR^−/low^CD14^+^ M-MDSC occur at higher frequency in peripheral blood of chronic HBV-infected patients and suppress HBV-specific CD8 T-cell cytotoxicity (105). Suppressive MDSC are also increased in murine HBV infection (45) and drive CD8 T-cell exhaustion *via* their crosstalk with γδT-cells (46). M-MDSC accumulate during viral coinfections, but frequencies appear to be similar with those observed in mono-infections (103). E.g., elevated number of MDSC were reported for HCV/HIV (103) and shown to regulate excessive IFN-γ production in HIV/CMV coinfected individuals (96).

#### Bacteria

Bacterial infections are often associated with excessive inflammation or low-grade chronic production of pro-inflammatory cytokines and chemokines known to induce the expansion and activation of MDSC. E.g., chronic *S. aureus* infection in mice is sustained by M-MDSC and PMN-MDSC expressing ARG1, iNOS, and IL-10 which foster an immunosuppressive environment and impair monocyte/macrophage responsiveness (33, 47, 48, 108). Similarly, during infections with intracellular bacteria, such as *F. tularensis*, MDSC frequencies correlate with the extent of tissue pathology, loss of pulmonary function, and host mortality (42). Several reports demonstrate that inoculation of mice with BCG or infection with *M. tuberculosis* induce M-MDSC that diminish pathogen control and promote disease lethality (50, 65, 109). Obregón-Henao provided new evidence, demonstrating accumulation of ARG1-producing MDSC in *M. tuberculosis*-infected mice (41). Similar findings were reported in human TB, with increased immunosuppressive M-MDSC in TB patients and individuals with recent exposure to TB patients (28, 110). More recently, a protective role of M-MDSC in early stages of BCG-induced pleurisy was reported (80). This effect has been linked to TNF-dependent suppression of CD4+ T-cell inflammation. MDSC were also highly induced following infection with a clinical isolate of multidrug-resistant *K. pneumoniae*. These M-MDSC express anti-inflammatory surface markers and displayed compromised phagocytic abilities (3). Impairment of IL-10 production from total MDSC inhibited resolution of *K. pneumoniae*-induced inflammation (4). *H. pylori*-mediated inflammation of the gastric mucosa also promoted an influx of M-MDSC that countered host protective TH1 immune responses (111). In addition, MDSC gradually increase after polymicrobial sepsis (75–77), with M-MDSC mainly promoting sepsis-induced mortality early during infection (75).

#### Fungi

TH17-polarized immunity is generally required for protection against fungal infections; however, fungi modulate host immunity by inducing immunosuppressive MDSC which could also benefit the host by reducing hyperinflammatory responses (84). The majority of studies only report the induction of PMN-MDSC following infection with pathogenic fungi, such as *Candida albicans* and *Aspergillus fumigatus* (84, 162). In line with this, treatment of mice with yeast-derived antigens, such as β-glucan specific to dectin-1, reduced accumulation of PMN-MDSC but not M-MDSC and significantly decreased tumor burden (163).

#### Protozoa

Induction of potent TH1 immunity is generally sufficient to protect the host against debilitating protozoal expansion and pathology. While MDSC are typically detrimental to diseases requiring a robust host protective TH1 response, MDSC induction could in fact be beneficial during infections triggering inflammation-mediated tissue damage. For example, chronic and acute protozoan infections with *L. major* or *Trypanosoma cruzi*, mediate induction of M-MDSC which protect against pathology and parasite load, despite suppression of T-cell proliferation (73, 116, 118), although contradictory evidence have been reported (117). Similar results were shown in a mouse model of *Toxoplasma gondii* infection, where the total MDSC population induced hyporesponsiveness and were required for resistance against the pathogen (119). Corroborating work demonstrated that the absence of cells resembling total MDSC during acute *T. gondii* infection resulted in extensive intestinal necrosis due to the host TH1 inflammatory response (119, 120). More recent data on *L. donovani* provided evidence of the expansion of myeloid cells, likely a combination of M-MDSC and PMN-MDSC, in the spleens of infected BALB/c and C57BL/6 mice. These cells exhibit TH1 immunosuppressive features and their immunosuppressive capacity is reduced following soluble leishmanial antigen vaccination (114, 115).

#### Helminths

Helminths characteristically cause stable, long-term infections with severe host immunomodulatory consequences, such as triggering TH2 host immune polarization. Several helminth species and their excretory/secretory products induce accumulation of M-MDSC, including *Schistosoma* spp. (121), *Echinnococcus granulosus* (122), and *Nippostrongylus brasiliensis* (123). Important work in a mouse model of *Heligmosomoides polygyrus bakeri* infection revealed the induction of a MDSC subset, likely comprising M-MDSC and PMN-MDSC, with TH2 immunosuppressive capabilities that exacerbate infection and worm burden (124, 125). Another important consideration during helminth infections is the host protective effect of MDSC-mediated suppression of TH1 immunity and induction of TH2 immunity. E.g., MDSC mediate enhanced pathogen clearance in a model of *N. brasiliensis* infection, although this appears to be specific to the granulocytic subset and might increase host susceptibility to diseases requiring TH1 for protection (123).

Monocytic myeloid-derived suppressor cells have been investigated only in a number of infections. In some circumstances, this MRC subset emerges as a regulator of disease pathogenesis. Based on depletion studies in animal models and correlative studies in humans undergoing anti-infective therapy, M-MDSC have both host-destructive and -protective roles. They promote establishment and progression of HIV/SIV (24, 25, 49), LCMV (71), staphylococcal prosthetic complications (33, 48, 108), and TB (29, 30) (Table 1). On the contrary, several studies indicate that this MRC subset protects from immunopathology, particularly in certain acute bacterial infections (35) and in protozoal infection (73), but also at distinct stages of viral infection with vaccinia virus (164). In such circumstances, M-MDSC contribute to resolution of inflammation or prevent disease flares. Such dual roles may correlate with biology of M-MDSC, notably their interaction with pathogens.

### Phagocytic M-MDSC Harboring Pathogens

Subcellular compartmentalization of microbes within M-MDSC, as well as how pathogens modulate cell death patterns or metabolic features of these monocytic cells have not been fully elucidated. Since MDSC are phagocytes, an alternative function of M-MDSC is as a reservoir for invading pathogens. Initial evidence of impaired pathogen elimination came from a mouse model showing that mycobacteria, notably BCG, are phagocytosed by CD11b^+^Ly6C^int^Ly6G^−^ MDSC (65). Despite NO production, they were unable to kill *M. bovis* or the nonpathogenic *M. smegmatis* and suppressed T-cell activation. More recent data demonstrate that murine MDSC, induced following *M. tuberculosis* infection, display dose-dependent phagocytic and endocytic capabilities (30). Considering that *M. tuberculosis* survival in phagocytes is attributed to host-derived lipids, and since these serve as their primary carbon source *via* the glyoxylate shunt, it is tempting to speculate that MDSC provide niche for pathogen persistence. This assumption is supported by the finding that MDSC highly express complement receptor-3 CD11b and receptors for oxidized lipid (oxLDL)-uptake (CD36 and LOX-1) (165), which assist *M. tuberculosis* engulfment (166, 167). MDSC-resembling cells were shown to contain microbes, such as *Escherichia coli* and *L. major* (52, 55, 73, 113).

Other investigators report on defects in MDSC phagocytic potential under conditions of persistent stimulation or chronic inflammation (168). M-MDSC displayed reduced uptake of *F. tularensis* in comparison to naïve bone marrow-derived macrophages or AM (42) and poor phagocytic/killing potential of *K. pneumoniae* (3). MDSC may also impair the phagocytic potential of other innate cells. For example, the phagocytic ability of AM is significantly reduced in the presence of MDSC from PcP-infected mice. These adverse effects on AM are dependent on MDSC expressing PD-L1 and induction of PD-1 expression in AM during PcP infection (153, 169). Nonetheless, others failed to show any significant impact of MDSC on macrophage phagocytic potential (170).

Besides harboring bacterial pathogens, M-MDSC may support replication of viruses. Retroviruses, including SIV (25), LP-BM5 (93), and HIV (24) have been detected within this monocytic subset in macaques, mice, and humans, respectively. M-MDSC may traffic and interact with lymphocytes and thereby contribute to viral spread, besides limiting functionality of T lymphocytes.

## Conclusion and Outlook

Many open questions and challenges for MDSC research remain. In particular, evidence on human MDSC subset characterization and their place in the spectrum of the myeloid lineage are still conflicting. In mice, TAM differentiation from M-MDSC may be accomplished to some extent based on positivity of TAM for F4/80 and their low or negative expression of Ly6C along with higher transcript levels for IRF8, M-CSF, and reduced ER-stress markers (2, 36, 171, 172). A detailed comparison between activated tissue macrophages and M-MDSC has not been conclusively conducted in infection. Lineage-tagging studies and phenotype stability are currently lacking and, therefore, tracing M-MDSC development in infection is either hypothetical or based on *ex vivo* observations and extrapolations from cancer models. Furthermore, a detailed understanding of the pathogen- and host-derived signals modulating MDSC induction and function will assist in the development of their therapeutic application. Specifically, the factors mediating suppression of host immunity in an antigen-specific manner need to be better understood to exploit drugs inhibiting MDSC in infections where these cells favor pathogen survival or limit optimal host responses. Moreover, pathogen responses, including stress and adaptation, to M-MDSC have not been investigated yet.

Although several therapeutic approaches involving re-purposed agents, mostly all-trans retinoic acid, effectively reverse MDSC immunosuppressive features in murine infection models of TB (30) and sepsis (173) as well as in few *ex vivo* human studies in HBV (18), comprehensive human clinical studies are required to systematically assess the safety, efficacy, dose, and timing of such interventions. Same rationale may improve vaccination in case of live vaccine, notably BCG and viral vector-based vaccines against HIV, known to trigger M-MDSC (65, 94). Furthermore, considering the diagnostic and prognostic potential of MDSC in the cancer field, these myeloid regulatory subsets should be considered for their potential role in biomarker development for infectious diseases.

## Author Contributions

All authors listed have made a substantial, direct, and intellectual contribution to the work, and approved it for publication.

## Conflict of Interest Statement

The authors declare that the research was conducted in the absence of any commercial or financial relationships that could be construed as a potential conflict of interest.
